# Supplementary material for: Planning with emission models reduces the carbon footprint of new reservoirs
Source: Commun Earth Environ. 2025 Nov 24;6(1):953. doi: 10.1038/s43247-025-02899-6 (PMC12646111; doi:10.1038/s43247-025-02899-6)
Supplement: Supplementary file 2 — Supplementary Information [file 43247_2025_2899_MOESM2_ESM.pdf]

Planning with emission models reduces the carbon footprint of new reservoirs

Tomasz Janus [1*], Christopher Barry [2], Shelly Win [3], Jaise Kuriakose [1*]

[1] Tyndall Centre Manchester, University of Manchester, Floor 5, Engineering A, Booth Street East, Manchester, M13 9PL, United Kingdom .
[2] UK Centre for Ecology & Hydrology, Environment Centre Wales, Bangor, United Kingdom .
[3] International Water Management Institute (IWMI), Myanmar Office, C/o Irrigation Head Office Yangon Compound, Thitsar Road, Yankin, Yangon, P.O. 11081, Myanmar .

*Corresponding author(s). E-mail(s): tomasz.janus@manchester.ac.uk; jaise.kuriakose@manchester.ac.uk;
Contributing authors: cbarry@ceh.ac.uk; s.win@cgiar.org;

1 Supplementary Information

1.1 Supplementary Notes

1.1.1 Supplementary Note 1 - Explainable AI and Sensitivity Analysis – Similarities, Differences and Prospects for Synergistic Applications

In this study, we employ explainable AI (xAI) methodology to enhance our understanding of the underlying drivers of predictions in the emission model. Explainable AI is a discipline related to sensitivity analysis (SA). Both disciplines aim to uncover relationships between inputs and outputs in complex systems and share a fundamental goal: enhancing transparency and interpretability. SA achieves this by analyzing model behaviour within mathematical and computational frameworks, while xAI provides human-interpretable insights into data-driven, often black-box, models [1].

Although both disciplines originate from two different traditions, i.e. mathematical modelling and machine learning, the increasing convergence of data-driven and model-driven approaches in science and engineering is gradually blurring the boundaries between these fields. Clear parallels can be drawn between xAI and SA methodologies. Both rely on perturbative and decomposition techniques to identify the importance of input features or parameters, [1]. For instance, global SA methods, such as variance-based approaches, share conceptual similarities with feature importance measures in xAI, including Shapley values [2] and LIME, [3]. Both frameworks often employ sampling or surrogate modelling to estimate the influence of inputs on outputs. Additionally, both aim to inform decision-making processes - whether it be model refinement in SA or actionable insights in xAI.

However, both fields are distinguished by their focus and application. SA traditionally centres on mathematical models, leveraging analytical tools to explore parameter space and diagnose models [4]. In contrast, xAI is inherently data-driven and primarily targets the interpretation of neural networks and other machine learning models, [2, 5]. SA is rooted in theoretical and often deterministic paradigms, while xAI adapts to stochastic and empirical frameworks. Moreover, xAI emphasizes user-centred explanations, ensuring that model predictions are interpretable to non-experts, whereas SA focuses on understanding models from a mathematical perspective. Finally, SA and xAI address different yet complementary aspects of model analysis. SA

is primarily used in scientific research to explore causal relationships, reduce dimensionality, quantify uncertainties, design experiments, and support decision-making. In contrast, xAI is designed to make black-box models more interpretable to non-experts in a clear and intuitive manner [6].

Large mathematical models, such as models of environmental systems, have become complex enough that they are difficult to comprehend even by the experts, effectively rendering them black-boxes despite being formulated with mathematical equations. Consequently, expert interpretation would typically be needed to translate outputs of such models into a form understandable enough to inform policy decisions. This capability positions xAI to play an increasing role in mathematical modelling beyond purely data-driven approaches. By adopting xAI, we can overcome certain limitations intrinsic to SA approaches in mathematical modelling.

For instance, many global SA methods require explicit formulation of probability distributions of inputs, a challenge when reliable data is sparse or subjective judgments are involved. xAI techniques like SHAP [2] or iBreakDown [7] do not depend on predefined input distributions, potentially bypassing the need for exhaustive statistical input specification. Traditional SA often generates statistical indices, such as Sobol' indices, which, while mathematically robust, can be abstract and less accessible to non-specialist stakeholders. In contrast, xAI provides intuitive, human-friendly explanations that effectively communicate complex relationships to diverse audiences, including policymakers and interdisciplinary teams. Additionally, xAI methods, such as SHAP or iBreakDown [7], effectively capture and explain input interactions and support categorical inputs, while traditional SA frameworks, albeit capable of assessing interaction effects (e.g., via higher-order Sobol' indices), often do so at a considerable computational cost.

Expanding xAI applications beyond machine learning into mathematical modelling aligns with the broader goal of fostering transparency and trust in scientific and engineering decision-making, as demonstrated by existing guidelines [8] and publications advocating for model transparency [9]. Integrating xAI techniques into complex environmental models could, for example, help practitioners combine rigorous uncertainty quantification via SA with user-friendly explanations enhancing the accessibility and usability of such models in decision-making contexts. An example of such a complex environmental model is the G-res emissions model instrumental to this discourse.

1.1.2 Supplementary Note 2 - Surrogate Models for Sensitivity Analysis and Explainable AI

Surrogate models, also known as response surface models, metamodels, or emulators [10], are simplified approximations of computationally intensive or obscure (black-box) models that are faster to compute, compatible with formal mathematical frameworks (e.g., for controller design), and easier to analyze and explain (e.g., for sensitivity analysis and interpretability). Surrogate modelling has become integral to SA, particularly in variance-based global sensitivity methods which require many model evaluations to compute sensitivity indices [11]. In some applications, the use of surrogate models in SA is not merely advantageous but essential due to prohibitive computational requirements imposed by the original model [12].

Surrogate models play a central role in many xAI methodologies which rely on approximating black-box models with more transparent and interpretable model structures. In fact, local surrogate modelling in the vicinity of an explanation is embedded directly within popular xAI algorithms such as SHAP [2] and LIME [13]. While many xAI methods are model-agnostic and capable of explaining any black-box model, they can have a substantial numerical burden, particularly for models with numerous features and observations. For instance, SHAP and similar methods like iBreakDown [7], which rely on evaluating model performance across combinatorially permuted sets of inputs, often require extensive numbers of model evaluations.

Adopting surrogate models in xAI not only typically leads to shorter execution times but also enables the development of more efficient algorithmic designs which leverage their interpretable structures. For instance, KernelSHAP [2] - a model-agnostic version of the SHAP algorithm - has an exponential complexity with respect to the number of input features. In contrast, alternative methods such as TreeSHAP [14], designed specifically for tree-structured models, exhibit low-order polynomial complexity relative to the tree characteristics. Approximating a complex black-box model with many features using a tree-structured surrogate, such as a random forest or gradient-boosted tree, can lead to substantial computational savings. These savings arise not only from reduced execution times but also from compatibility with more efficient xAI algorithms.

Finally, since xAI primarily targets machine learning applications, opting for a surrogate-based data-driven approach replacing a generic black-box model can greatly simplify the analysis by eliminating the need to write custom interfaces between the model and the xAI algorithms. This approach also streamlines the implementation of preprocessing steps, such as removal of unwanted or correlated features, creating composite features, and feature scaling, all of which can be easily handled within established machine learning (ML)

frameworks without requiring custom code. However, substituting original models with surrogates may result in a loss of accuracy, potentially compromising the quality of outcomes from both SA and xAI frameworks.

Surrogate-based approaches therefore face the dual challenge of managing the additional uncertainty introduced by the surrogate model while ensuring that surrogate-driven results stay within acceptable error bounds. Furthermore, xAI applications additionally demand that model results are not only accurate but also faithful - meaning that the explanations generated by the surrogate model accurately reflect the reasoning of the original model. While faithfulness is critical to deriving reliable and actionable insights, it can be challenging to prove that a surrogate model faithfully represents the original.

On the other hand, faithfulness may not always be guaranteed, even when xAI is applied directly to the original model [15], due to factors such as correlated features or degrees of non-linearity that some xAI feature attribution methods, like SHAP [2] or LIME [13], cannot effectively handle. Therefore, it is theoretically possible that explanations derived via a surrogate model with a more interpretable structure and having passed appropriate pre-processing and feature engineering steps, can yield more faithful interpretations than when based on the original model. While xAI is rapidly advancing, there is still no consensus on a universally optimal approach to model interpretability. Finding optimal trade-offs between interpretability, accuracy, faithfulness, computational cost and other important model characteristics, remain an active area of research [16].

1.2 Supplementary Results

1.2.1 Emissions from installed and planned hydropower

We explored areal emissions of hydroelectric and energy-generating multipurpose reservoirs together with their annual generation estimates to determine emission intensities of existing and planned (future) hydropower in Myanmar. This provides the foundation for the strategic investment planning of hydroelectric assets using greenhouse gas (GHG) emissions as one of the selection criteria, and notably illustrates the large variability in the hydroelectric reservoirs emission intensities.

Supplementary Figure 2a visualizes differences in annual power generation and emission intensities of existing and planned (future) hydropower across the country. Current hydropower generation is concentrated in 11 run-of-river (RoR) and 18 reservoir-based hydroelectric plants (6 hydroelectric and 12 multipurpose) situated at low altitudes and having high emission intensities. The bulk of the energy is generated in multipurpose dams such as Thapanseik, Zawgyi II, and Sedawgyi (Supplementary Figure 2b), with respective emission intensities of 2,207, 1,649, and 509 gCO_{2e}/kWh. These emission intensities are comparable to those of coal- (median lifecycle emissions: 820 gCO_{2e}/kWh) and gas-powered plants (median lifecycle emissions: 490 gCO_{2e}/kWh) [17, Annex III Table A.III.2].

Most planned hydroelectric plants, including storage dams and RoR units, are situated in the eastern and northern parts of the country, primarily along the upper Irrawaddy and Salween rivers. Consequently, they have a lower mean emission intensity (52 gCO_{2e}/kWh) than the existing storage dams (270 gCO_{2e}/kWh). However, certain planned hydroelectric reservoirs like Lemro 2 and Belin exhibit high biogenic emission intensities (616 gCO_{2e}/kWh and 186 gCO_{2e}/kWh, respectively), higher than the reported median values of 63 gCO_{2e}/kWh [18] and 24 gCO_{2e}/kWh [17]. The wide variability in emission intensities of existing (3-1,657 gCO_{2e}/kWh) and future storage dams (0.5-194 gCO_{2e}/kWh) underscores the importance of accounting for emissions in reservoir planning.

1.2.2 Comparison of emission estimates: G-res vs. Emission Factors

A comparison of GHG emission predictions using the G-res methodology and global Tier 1 climate-zone-based emission factors (EFs) highlights the limitations of EF-based approaches in capturing the complexity of reservoir emissions. This is particularly evident for CH_4 emissions, which have more complex pathways compared to CO_2. The G-res methodology, with its detailed modelling, provides a more nuanced estimation of gross emissions and introduces mechanisms for disentangling net anthropogenic emissions from total (gross) emissions, which are otherwise treated simplistically - see Eq. 1.

Gross emission predictions from G-res and the Tier 1 emission factor (EF) method exhibit pronounced discrepancies in both variance and bias. Gross emissions estimated with the global Tier 1 emission factor (EF) [19] exceed those estimated with G-res - in particular CH_4 emissions of hydroelectric reservoirs; see Supplementary Fig. 4a. Most of the variance and bias stem from CH_4 diffusion and ebullition of hydroelectric dams; Supplementary Fig. 6. By recalibrating emission factors (see Methods) to match G-res outputs,

we reduced bias and variance for some emission pathways, such as CH_4 diffusion and CH_4 ebullition (Supplementary Fig. 4b, Supplementary Fig. 6). However, recalibration was less effective for other pathways, such as CH_4 degassing in hydroelectric reservoirs. This finding underscores the inability of simple GHG emission parametrizations, such as emission fluxes tied to climatic zones, to capture the complex dependencies between multiple emission drivers and resultant emissions.

Net anthropogenic emissions derived from Tier 1 gross estimates using Eq. 1 generally exceed those from G-res (Supplementary Fig. 7), although net CO_2 emissions are, on average, slightly underestimated. The agreement between the two methods is stronger for CO_2 emissions than for CH_4, with methane predictions exhibiting greater variance and bias – particularly among irrigation reservoirs, as shown in Supplementary Fig. 5a. Recalibrating the net emission coefficients in Eq. 1 to better align Tier 1 net emission estimates with G-res predictions improves the agreement between EF-derived and G-res-derived net emissions (Supplementary Fig. 5b). However, substantial discrepancies remain, with errors reaching up to 200% (Supplementary Fig. 7). While recalibration using linear regression reduced the total squared error – mainly driven by hydroelectric reservoirs – it also led to an overestimation of net_{CH_4} emissions in irrigation reservoirs. This outcome suggests that net_{CH_4} coefficients may not be uniform (e.g. for a region), but could vary across reservoir types.

The values of emission factors and net_{CO_2} and net_{CH_4} coefficients together with fitness score metrics measuring the level of fit between EF-based emission estimates and G-res outputs, are provided in the Manuscript and in Supplementary Tables: 6, and 7. The fit metrics in Supplementary Table 6 indicate an improved agreement between Tier 1 predictions and G-res estimates with the recalibrated emission factors, though prediction errors remain large - especially for CH_4 degassing, where calibration was ineffective, highlighting the inefficiency of EF-based approaches for this emission pathway. Calibration of net_{CO_2} and net_{CH_4} coefficients led to approximately a two-fold improvement in fit quality represented with mean absolute error (MAE) and root mean square error (RMSE) metrics. For net CO_2 emissions, the R^2 value increased from approximately 0.55 to over 0.9. However, for net CH_4 emissions, R^2 decreased despite improvements in MAE and RMSE, indicating poor alignment of data points with a single linear trend.

The calibrated net_{CH_4} values come with wide uncertainty bounds ($[0.157, 0.623]$ and $[0.167, 0.641]$, respectively), highlighting the difficulty of accurately deriving net CH_4 emission predictions from gross estimates using a single, universally applied proportionality constant across all reservoirs.

1.2.3 Similarities between asset portfolios for different emission estimation methods

Differences in net emissions from existing and planned hydroelectric reservoirs in Myanmar, calculated using G-res and global Tier 1 emission factors, are visualised in Supplementary Figures 5 and 7. Tier 1 net emission estimates are generally higher than those produced by the G-res method. At the level of individual reservoirs, relative errors can exceed 100%. These differences can alter asset portfolios selected strategic dam planning, depending on the choice of method for predicting GHG emissions.

We assessed the impact of the choice of emission model on asset selection using the mean Jaccard index (see Supplementary Methods), which quantifies the similarity between Pareto-optimal dam portfolios generated using GHG emissions estimated with two different methods. The similarity was evaluated across a range of hydropower (HP) generation targets, grouped into bins with a width of 0.121 TWh/year. The mean Jaccard index ranges from 0 (no overlap between portfolios) to 1 (perfect overlap). Figure 8 shows the mean Jaccard indices for Pareto-optimal dam portfolios calculated under two optimization scenarios – *Built* and *Not Built*.

The results in Supplementary Fig. 8 show greater overall similarities within the *Built* scenario compared to the *Not Built* scenario. In the *Not Built* scenario, the optimizer has access to more candidate assets that lead to a greater variety of asset combinations in the computed asset portfolios. Similarities are lower and exhibit greater variability in lower HP generation target ranges (shaded region). This is primarily due to portfolio size effects, where differences in individual reservoir selection have a proportionally larger impact on similarity for portfolios with fewer assets. Additionally, greater flexibility in early optimization stages, when more reservoirs are available as candidate solutions, contributes to this variability. For HP production targets above 50 TWh/year, the average similarity index is 0.88 for the *Built* scenario and 0.83 for the *Not Built* scenario. However, for certain HP production targets, these indices can drop as low as 0.7 and 0.6, respectively.

1.3 Supplementary Methods

1.3.1 Comparison of G-res and Tier 1 emission estimates

We compared emissions derived from Tier 1 emission factors with those produced using the G-res methodology. To visualize the similarities and differences between the two approaches, we used pair plots, where the emission outputs from G-res and those derived from emission factors were plotted on separate axes. Additionally, we quantified the fit between the outputs of both approaches using three metrics: the coefficient of determination (R^2), mean absolute error (MAE), and root mean square error (RMSE). R^2 evaluates the proportion of variation in the dependent variable explained by the independent variable, MAE measures the average magnitude of errors between two datasets, and RMSE assesses the error between the datasets while placing greater emphasis on larger errors. Together, these metrics provide complementary information quantifying the quality of fit between emissions modelled with G-res and approximated with Tier 1 emission factors.

We compared gross emission estimates from the G-res model with those derived using Tier 1 emission factors. We considered global emission factors published by Soued et al. [19] as well as calibrated emission factors obtained via linear regression on G-res input-output data. These calibrated factors can be regarded as country-specific Tier 1 emissions, using G-res predictions for upscaling. In addition, we computed net anthropogenic emission estimates using both approaches. Net emissions from Tier 1 gross emission factors were calculated using the linear equation proposed by Almeida et al. [20] (Eq. 1) and adopted in recent hydropower planning studies [21, 22] (see Methods).

1.3.2 Comparison of portfolio optimization results with different objective formulations

We measured the similarity between hydroelectric asset portfolios selected by the optimizer under different sets of objectives using the average Jaccard index, which quantifies the similarity between collections of sets. For any two collections of sets, $\mathcal{A} = \{\mathcal{A}_1, \mathcal{A}_2, \ldots, \mathcal{A}_n\}$ and $\mathcal{A} = \{\mathcal{B}_1, \mathcal{B}_2 \ldots, \mathcal{B}_m\}$ the average Jaccard index is calculated as:

$$\bar{J}(\mathcal{A}, \mathcal{B}) = \frac{1}{n \times m} \sum_{i=1}^{n} \sum_{j=1}^{m} J(\mathcal{A}_i, \mathcal{B}_j) \tag{1}$$

where $J(\mathcal{A}_i, \mathcal{B}_j)$ is the Jaccard index between sets $\mathcal{A}_i$ and $\mathcal{B}_j$.

The Jaccard index measures the similarity between two sets as the ratio of the number of items present in both sets to the total number of unique items in the two sets combined. Formally, and in the context of the equation above, the Jaccard index is defined as:

$$J(\mathcal{A}_i, \mathcal{B}_j) = \frac{|\mathcal{A}_i \cap \mathcal{B}_j|}{|\mathcal{A}_i \cup \mathcal{B}_j|} \tag{2}$$

where $|\mathcal{A}_i \cap \mathcal{B}_j|$ represents the cardinality of the intersection of sets $\mathcal{A}_i$ and $\mathcal{B}_j$, and $|A_i \cup \mathcal{B}_j|$ is the cardinality of their union.

We used the average Jaccard index to evaluate the similarity between asset portfolios selected by the five-objective optimization formulation when the GHG emissions objective was calculated using G-res (model) and estimated using Tier 1 emission factor (EF).

The Pareto fronts – one generated using G-res model predictions as the emissions objective and the other using emission factors for the emissions objective – were divided into 2,000 shared bins for both fronts. We then identified the sets of assets in each bin for each Pareto front and calculated the average Jaccard index for each bin. This analysis was repeated for two optimization scenarios: the *Built* scenario, which includes existing assets, and the *Not Built* scenario, which assumes no constructed assets.

1.4 Supplementary Figures

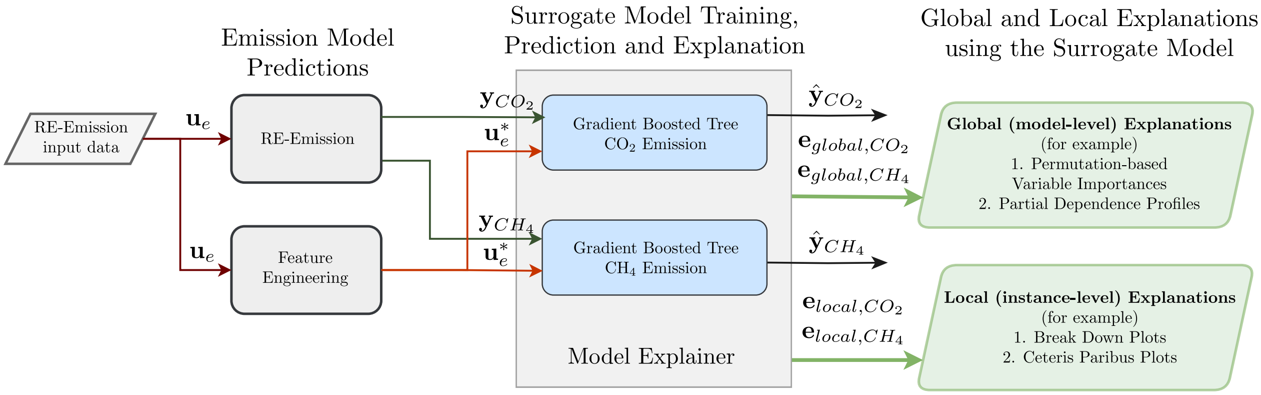

Supplementary Fig. 1: The workflow for generating model-level and instance-level explanations of emission predictions. Input data $\mathbf{u}_e$, produced by GeoCARET [23] are passed to the RE-Emission model [24], which outputs vectors of net areal CO_2 and CH_4 emissions ($\mathbf{y}_{CO_2}$ and $\mathbf{y}_{CH_4}$ respectively), where each element corresponds to a single reservoir. Surrogate models (one for each gas) are trained on input-output data pairs, where the inputs $\mathbf{u}_e^*$ are derived from $\mathbf{u}_e$ following feature engineering, and the outputs $\mathbf{y}_{(\cdot)}$ are the emission estimates from RE-Emission. The surrogate models, in the form of boosted decision trees, produce approximations $\hat{\mathbf{y}}_{CO_2}$ and $\hat{\mathbf{y}}_{CH_4}$ of the original RE-Emission outputs. The surrogate models are wrapped using the `Explainer` interface from the DALEX package [25, 26], enabling the extraction of both global (model-level) and local (instance-level) explanations of emission predictions ($\mathbf{e}$) based on the surrogate model predictions and the input-output data.

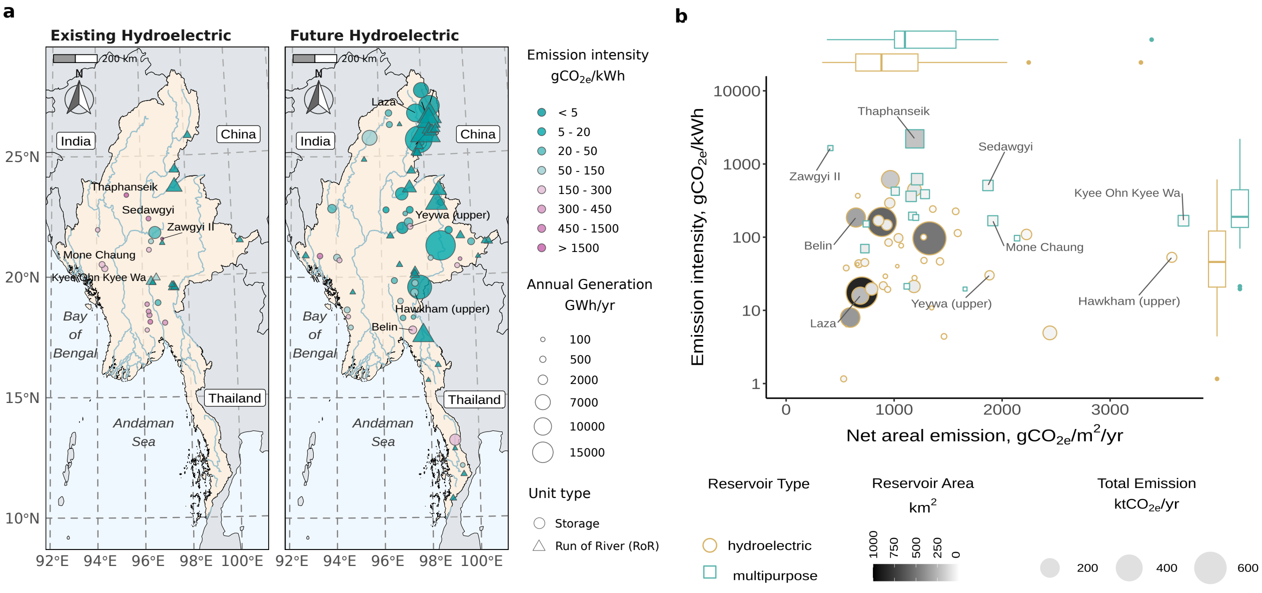

Supplementary Fig. 2: **Greenhouse gas emissions from hydroelectric power plants in Myanmar**. **a**, Reservoir icon size indicates projected annual hydropower generation, while colour represents the modelled emission intensity. Storage dams are shown as circles, and run-of-river (RoR) units as triangles. **b**, Emission intensities of hydroelectric (circles) and multipurpose (squares) storage dams plotted as a function of net areal emissions. Marker size is proportional to total annual emissions, and shading reflects reservoir area.

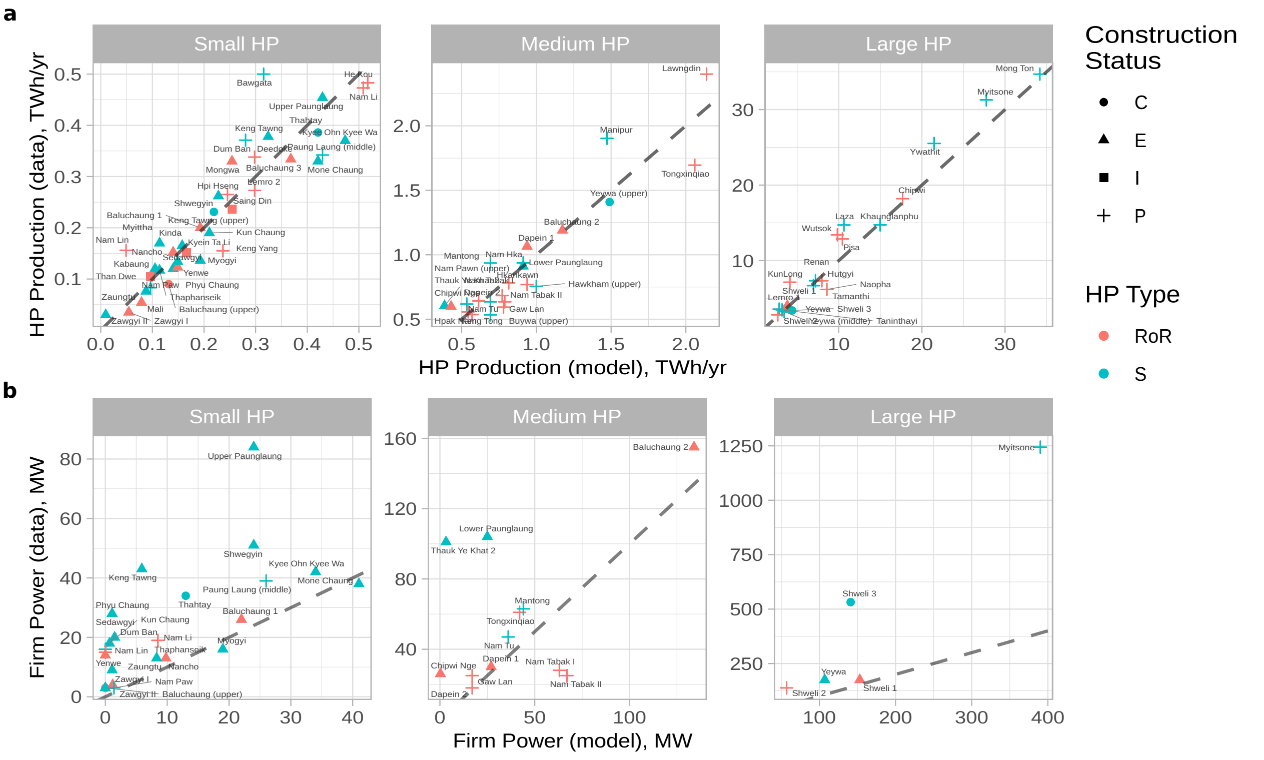

Supplementary Fig. 3: **Comparison of simulated hydropower generation with data from the IFC database of dams in Myanmar**. Simulated values from the national-scale water resources model are compared with reported data from the IFC database [27], for both mean annual hydropower (HP) generation (**a**) and firm power (**b**). Dashed lines indicate the 1:1 line of perfect agreement. Hydropower assets are classified by type – run-of-river (RoR) and storage (S) – and by development status: existing (E), under construction (C), identified (I), and planned (P). They are further grouped into Small HP (0-0.5 TWh/year), Medium HP (0.5-2.5 TWh/year), and Large HP (>2.5 TWh/year) categories based on annual generation capacity. **a**, Simulated mean annual generation closely matches IFC-reported values, with minimal bias and variance around the line of perfect fit. **b**, Simulated firm power estimates are consistently lower than those reported in the IFC database.

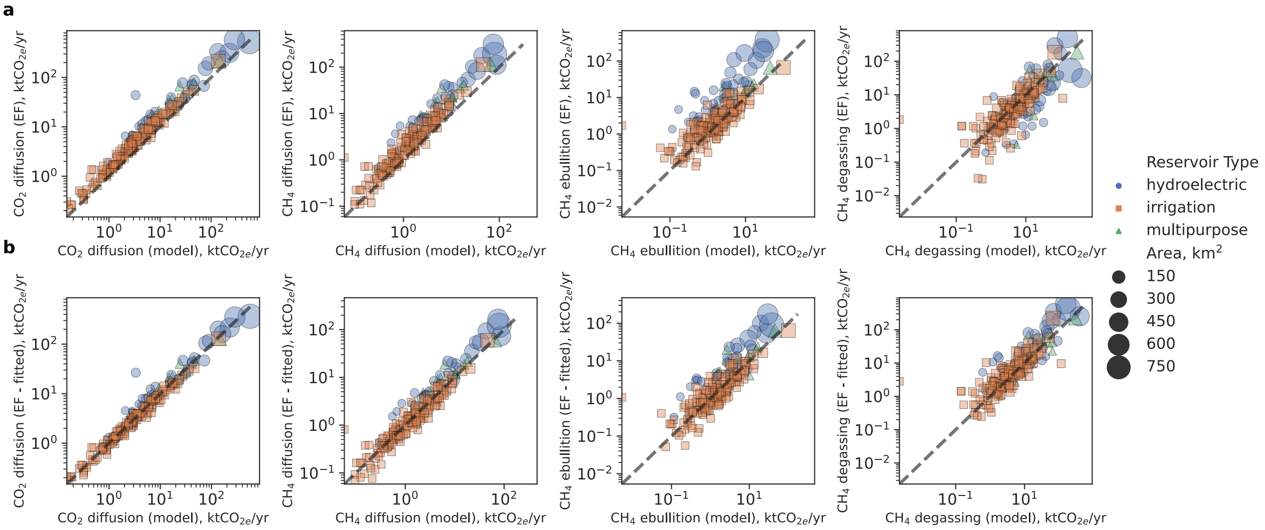

Supplementary Fig. 4: **Comparison of gross annual emission estimates from the G-res model and Tier 1 emission factors (EFs) for each individual emission pathway. a**, Comparison against global Tier 1 emission factors [19]. **b**, Comparison against Myanmar-specific emission factors derived via linear regression using G-res model outputs as the source of target values.

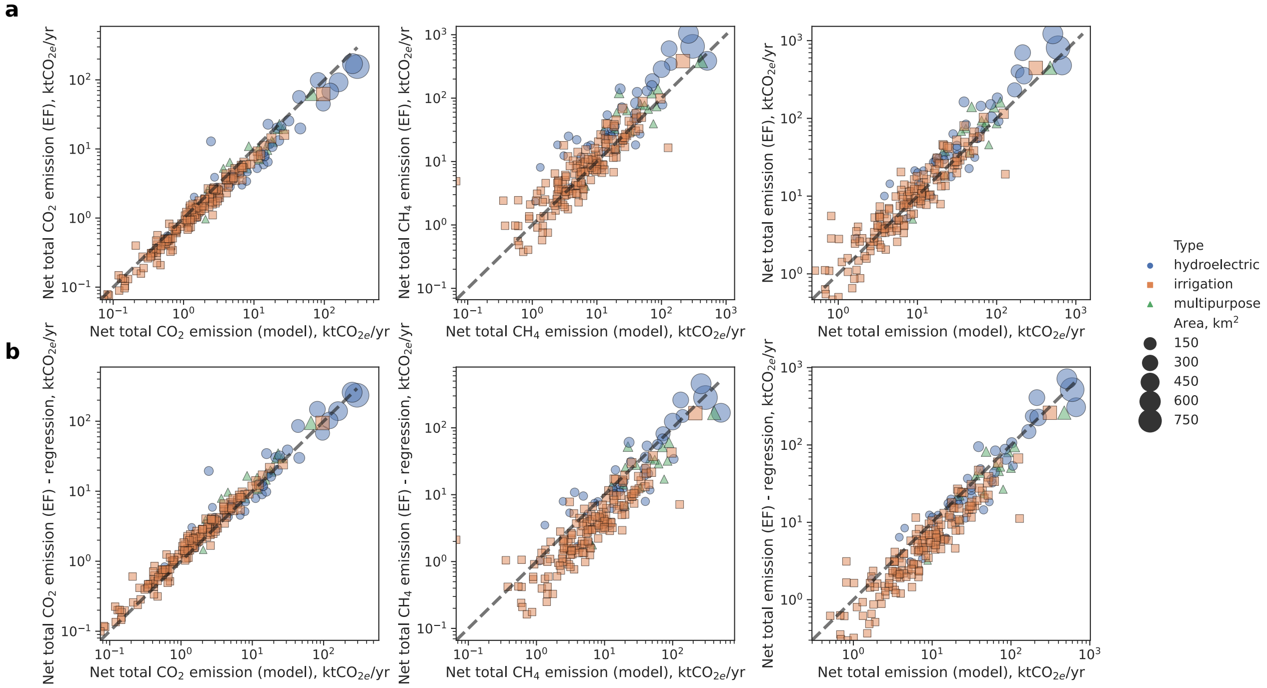

Supplementary Fig. 5: **Comparison of net total annual emission estimates from the G-res model and Tier 1 emission factors (EFs) for CO_2 and CH_4 individually and combined**. The net Tier 1 emissions are calculated from Tier 1 gross emissions using Equation 1, while the G-res methodology uses explicit calculations for net anthropogenic contribution to emissions. **a**, Net anthropogenic emissions derived from global Tier 1 emission factors [19] and Equation 1 with default regression coefficients [20]. **b**, Net anthropogenic emissions derived from global Tier 1 emission factors [19] and Equation 1 using regression coefficients optimized using linear regression minimizing the total squared error between Tier 1 (EF) and G-res (modelled) net emission predictions.

.

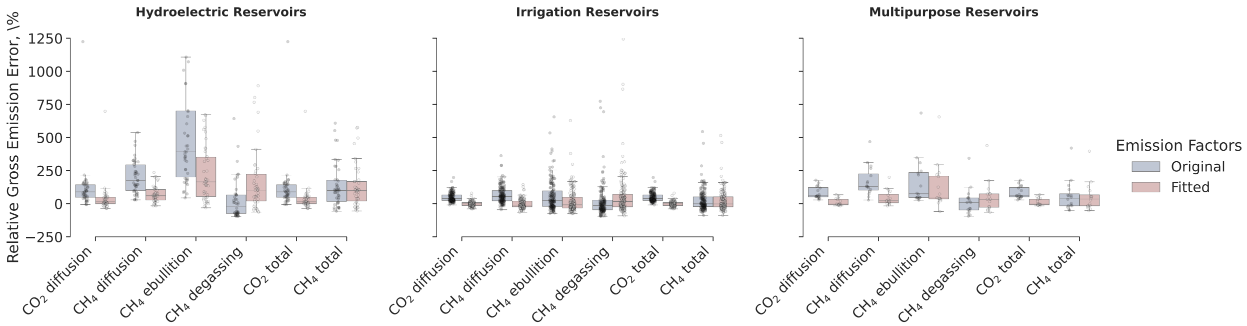

Supplementary Fig. 6: **Relative errors in gross emission estimates between the Tier 1 method and G-res, disaggregated by reservoir type and emission pathway.** Relative errors were calculated as $\mathrm{RE} = (\mathrm{TE}_{\mathrm{EF}}^{\mathrm{gross}} - \mathrm{TE}_{\mathrm{G\text{-}res}}^{\mathrm{gross}})/\mathrm{TE}_{\mathrm{G\text{-}res}}^{\mathrm{gross}} \times 100$, where $\mathrm{TE}_{\mathrm{EF}}^{\mathrm{gross}}$ denotes gross emissions derived from global Tier 1 emission factors, and $\mathrm{TE}_{\mathrm{G\text{-}res}}^{\mathrm{gross}}$ represents gross emissions from the G-res model. We used global Tier 1 emission factors [19] (Original) and Myanmar-specific emission factors calculated via linear regression using G-res predictions as target values (Fitted). Positive values indicate overestimation by the EF approach relative to G-res, and negative values indicate underestimation. To aid visual interpretation, the y-axis is capped at 1,250. However, relative errors for CH_4 ebullition in hydroelectric reservoirs exceed this limit, with the 95th and 99.7th percentiles reaching 2,570 and 3,083, respectively.

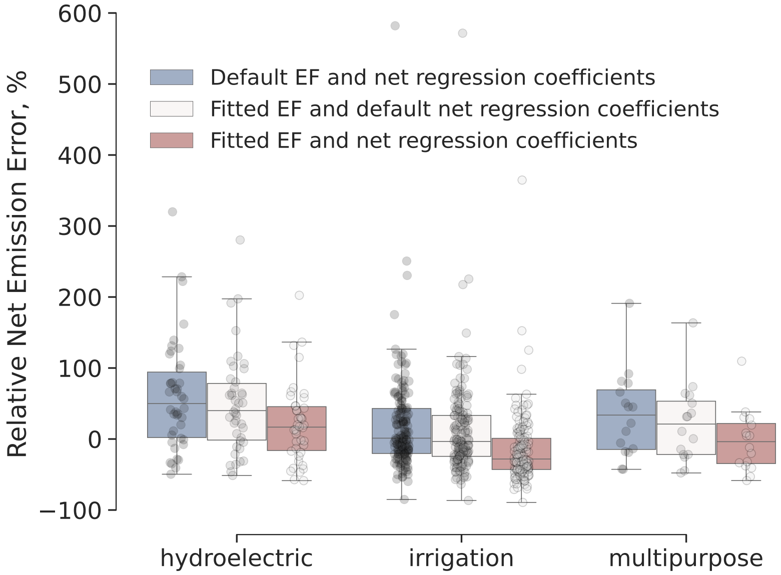

Supplementary Fig. 7: **Relative errors in total net emissions between the Tier 1 method and G-res.** Relative errors were calculated as RE $= \left(\mathrm{TE}^{\mathrm{net}}_{\mathrm{EF}} - \mathrm{TE}^{\mathrm{net}}_{\text{G-res}}\right)/\mathrm{TE}^{\mathrm{net}}_{\text{G-res}} \times 100$ where $\mathrm{TE}^{\mathrm{net}}_{\mathrm{EF}}$ denotes total net emissions derived using emission factors and $\mathrm{TE}^{\mathrm{net}}_{\text{G-res}}$ represents total net emissions calculated with the G-res model. Positive values indicate overestimation by the EF approach relative to G-res, and negative values indicate underestimation. Three scenarios are shown: (a) Global Tier 1 emission factors (Default) emission factors [19] and original (default) coefficients in Equation 1 [20], (b) Myanmar-specific emission factors calculated via linear regression to match the G-res outputs (Fitted) and original (default coefficients) coefficients in Equation 1, and (c) Both, emission factors and regression coefficients fitted via linear regression to the G-res outputs.

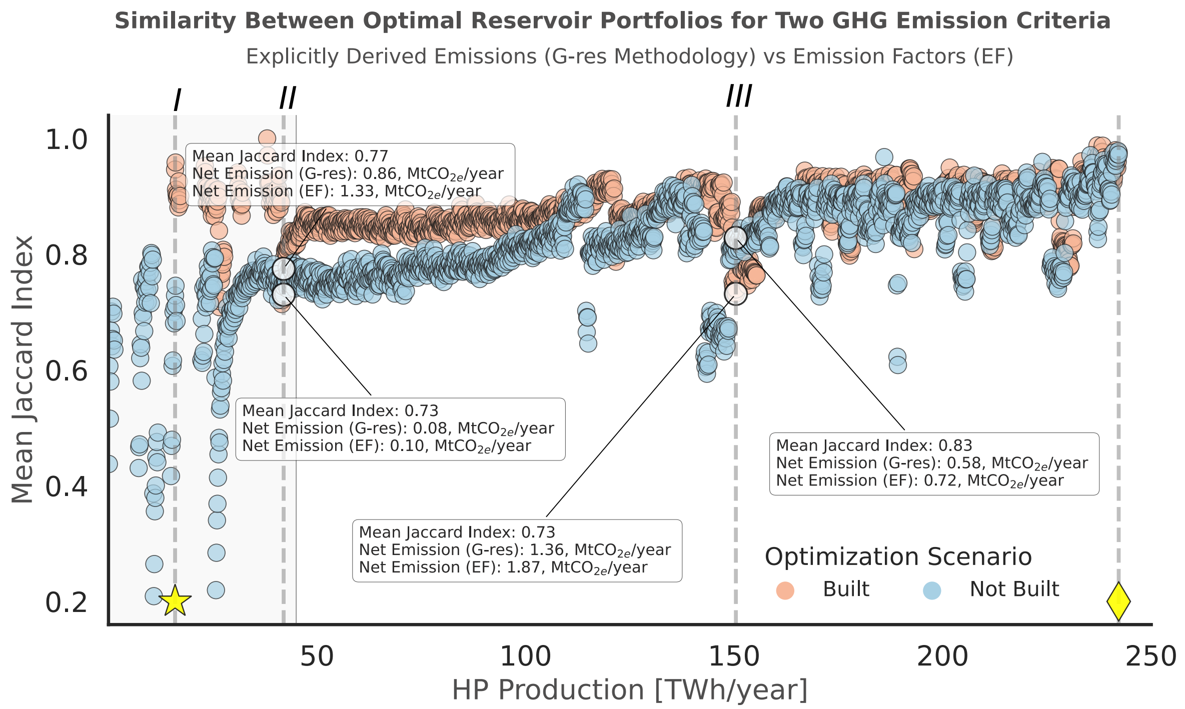

Supplementary Fig. 8: **Similarity between reservoir portfolios selected for two different sources of GHG emissions data.** Mean Jaccard indices quantify the similarity (ranging from 0 for no overlap to 1 for a perfect match) between dam portfolios selected by the optimizer when the GHG emission objective is determined using either the G-res model or global Tier 1 emission factors [19]. Values are shown across hydropower (HP) production targets, grouped into bins of width $0.121\,\mathrm{TWh\,yr^{-1}}$, for the *Built* and *Not Built* scenarios. The yellow star marks the current HP production from installed assets; the yellow diamond indicates the maximum achievable production using all existing and planned assets. The grey shaded region highlights areas of low portfolio similarity – particularly under the *Not Built* scenario. Annotated points *I*, *II*, and *III* correspond to selected solutions used in subsequent visualisations of optimal hydropower portfolios and their objective values.

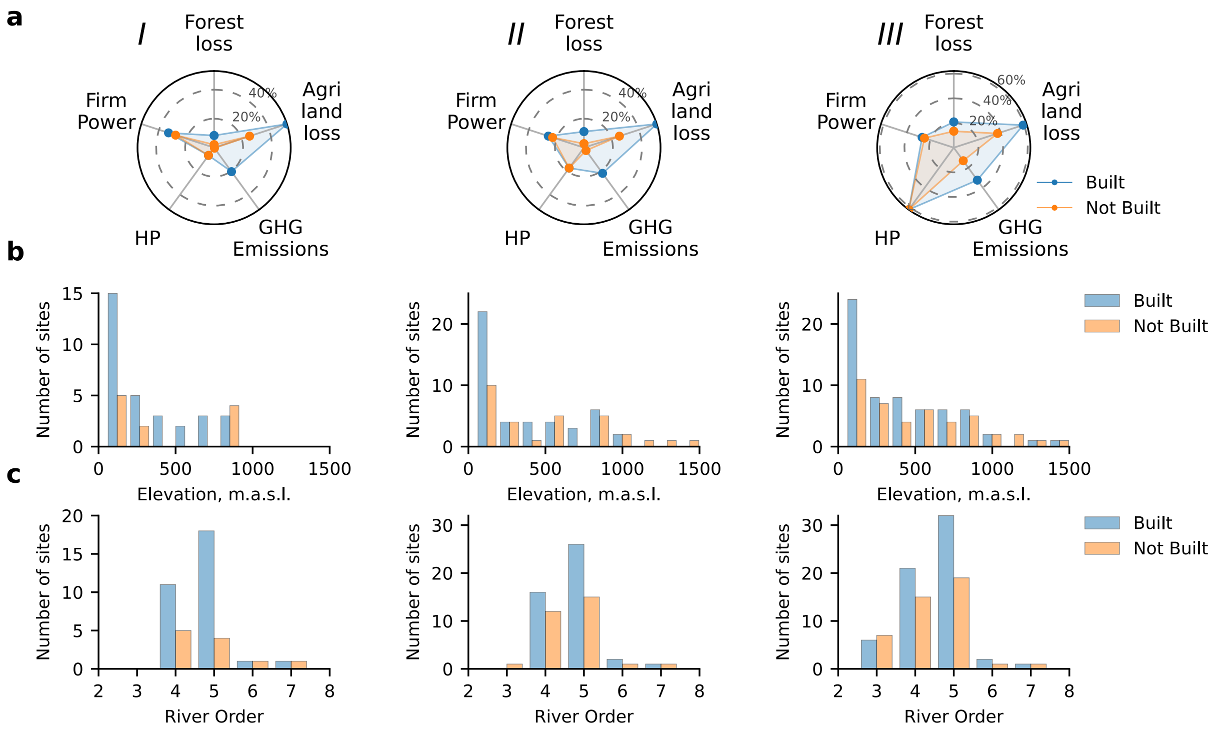

Supplementary Fig. 9: **Comparison of optimal hydroelectric dam planning solutions for built and not-built scenarios across three hydroelectric generation targets: I - current generation - 14.5 TWh/year, II - 42 TWh/year, III - 150 TWh/year using G-res emission outputs as one of the selection criteria.**. **a**, Radar plots displaying the relative values of five objectives: annual hydropower generation (HP), firm power, forest loss, agricultural land loss, and GHG emissions, normalized to the maximum objective value across all Pareto-optimal solutions. **b**, Distribution of hydroelectric assets by water table elevation at full supply level. **c**, Distribution of hydroelectric assets by river order, as defined in HydroSHEDS [28]. River order values (1 to 10) are are calculated based on average river discharge in m^3/s using a logarithmic progression: *1 = >100000 *2 = 10000–100000 *3 = 1000–10000, ...

.

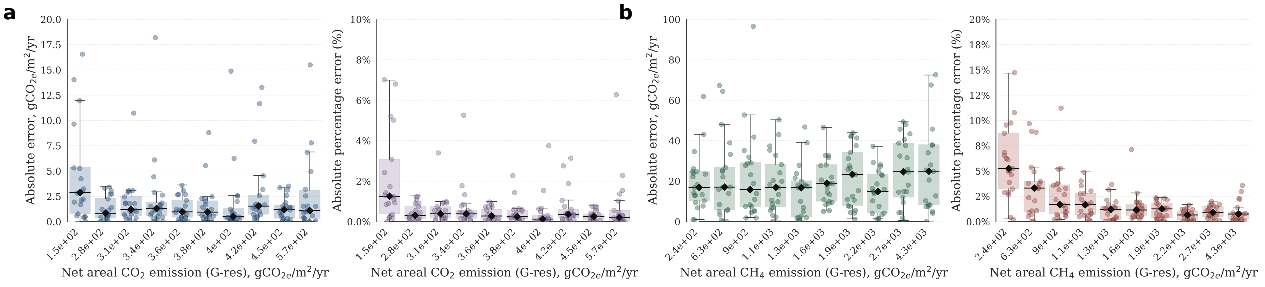

Supplementary Fig. 10: **Distributions of prediction errors for net areal greenhouse gas emissions predicted with a LightGBM surrogate model, expressed relative to G-res model estimates.** **a**, Sample-wise absolute errors (left) and absolute percentage errors (right) for net areal CO_2 emissions, grouped by emission quantiles. **b**, Same as in (a), but for net areal CH_4 emissions. Boxplots show the distribution of errors, black diamonds denote per-bin medians, boxes the interquartile range, whiskers 1.5 times the interquartile range (IQR), and points the individual errors.

1.5 Supplementary Tables

Supplementary Table 1: Statistics of the existing and future (under-construction, identified and planned) hydroelectric, irrigation and multipurpose reservoirs in Myanmar with respect to impoundment area, volume, and mean depth and areal emissions calculated with G-res.

Type	Hydroelectric			Irrigation			Multipurpose		
	min	max	median	min	max	median	min	max	median
Area, km^2	3.05	853	29.2	0.208	54	3.64	5.28	211	37.8
Volume, km^3	0.104	56.9	0.985	0.000223	0.894	0.0201	0.142	4.25	0.602
Mean depth, m	13.9	85.1	43.4	0.4	24	5.8	7.2	64.1	15.5
Areal CO_2 emission, $gCO_{2e}/m^2/yr$	56.6	673	329	156	671	376	209	482	333
Areal CH_4 emission, $gCO_{2e}/m^2/yr$	112	3,136	634	31.4	14,029	1,748	333	3,469	1,118
Total areal emission, $gCO_{2e}/m^2/yr$	363	3,571	954	386	14,275	2,115	729	3,679	1,461

Supplementary Table 2: Summary statistics of existing and future (identified, under-construction, and planned) storage and run-of-river hydropower projects in Myanmar, including annual emissions, impoundment area, storage volume, and mean depth.

Type	n	Emissions, $ktCO_{2e}/yr$		Area, km^2	Volume, km^3	Mean depth, m
		G-Res	EF[1]			
			**Existing reservoirs**			
Hydroelectric[2]	5	289.2	541.4	255.2	7.14	26.3
Irrigation	152	2,336	2,570	1,178	7.97	6.46
Multipurpose-hp	13	844.0	932.5	653.3	8.57	17.0
Multipurpose-irr		369.5	408.2			
RoR	13	27.43[3]		–	–	–
			**Future reservoirs**			
Hydroelectric	37	3,456	5,372	3,921	175.6	36.05
Irrigation	0	0.0	0.0	0.0	0.0	n/a
Multipurpose-hp	3	97.9	219.7	106.4	5.39	39.9
Multipurpose-irr		0[4]	0[4]			
RoR	35	231.3[2]		–	–	–

[1]Calculated using global Tier 1 emission factors per climatic zone and emission pathway [19].

[2]Keng Tawng, originally listed as a hydroelectric reservoir in the IFC database is considered here as RoR as its area, according to IFC, is 0.1 km^2, i.e. below the delineation threshold value of 0.5 km^2 adopted in this study.

[3]Emissions of RoR hydroelectric generation plants were calculated from the hydroelectric generation estimates calculated in the water resources model and assuming a GHG emission intensity of 3 gCO_{2e}/kWh across RoR generators.

[4]Emissions from future multipurpose reservoirs were attributed entirely to hydropower due to lack of information about future irrigation expansion plans.

Supplementary Table 3: Quantitative evaluation of LightGBM surrogate models for net areal CO_2 and CH_4 emissions across the full dataset. Reported metrics include root mean squared error (RMSE), mean absolute error (MAE), their relative forms (RRMSE and RMAE, expressed as percentages of the mean observed value $\bar{y}$), and the coefficient of determination (R^2).

Model	RMSE ($gCO_{2e}/m^2/yr$)	RRMSE (%)	MAE ($gCO_{2e}/m^2/yr$)	RMAE (%)	R^2
Net areal CO_2 emission	6.21	1.69	2.55	0.70	0.995
Net areal CH_4 emission	31.3	1.92	21.9	1.34	0.999

Supplementary Table 4: Geospatial datasets (assets) used by GeoCARET for reservoir and catchment-scale analyses.

No.	Asset Name	GEE Data URL	Website	References
		Google Earth Engine (Public) Assets		
1	WWF Hydrosheds[3]	WWF/HydroSHEDS	gee1, gee2, original source	[28]
2	WWF Hydrosheds Flow Accumulation 15 Arc Seconds	WWF/HydroSHEDS/15ACC	gee, original source	[28]
3	WWF Hydrosheds Drainage Direction 15 Arc Seconds	WWF/HydroSHEDS/15DIR	gee, original source	[28]
4	Hydrobasins 12	WWF/HydroSHEDS/v1/Basins/hybas_12	gee, original source	[28]
5	NASA SRTM Digital Elevation 30m	USGS/SRTMGL1_003	gee, original source	[29]
7	RESOLVE Ecoregions 2017	RESOLVE/ECOREGIONS/2017	gee, original source	[30]
8	Soilgrids 250 m OCS	projects/soilgrids-isric/ocs_mean	gee	[31]
9	Soilgrids 250 m SOC	projects/soilgrids-isric/soc_mean	gee	[31]
10	Soilgrids 250 m N	projects/soilgrids-isric/nitrogen_mean	gee	[31]
11	Soilgrids 250 m BDOD	projects/soilgrids-isric/bdod_mean	gee	[31]
12	Monthly Climate and Climatic Water Balance for Global Terrestrial Surfaces, University of Idaho	IDAHO_EPSCOR/TERRACLIMATE	gee, original source	[32]
13	NASA-USDA Enhanced SMAP Global Soil Moisture Data	NASA_USDA/HSL/SMAP10KM_soil_moisture	gee, original source	[33]
14	GPWv411: Population Density (Gridded Population of the World Version 4.11)	CIESIN/GPWv411/GPW_Population_Density	gee, original source	[34]
		Assets Imported to Google Earth Engine from External Sources[2]		
15	Average Monthly and Annual Direct Normal Irradiance Data, One-Degree Resolution of the World, NASA/SSE, 1983-2005	[home-folder]/XHEET_ASSETS/GHI_NASA_low	original source	[35]
16	UNH-GRDC Composite Runoff Fields V1.0	[home-folder]/XHEET_ASSETS/cmp_ro_grdc	original source	[36]
17	WorldClim 2.1: new 1-km spatial resolution climate surfaces for global land areas (monthly mean temperature variables)	[home-folder]/XHEET_ASSETS/wc2-1_30s_tavg	original source	[37]
18	WorldClim 2.1: new 1-km spatial resolution climate surfaces for global land areas (bioclimactic variables)	[home-folder]/XHEET_ASSETS/wc2-1_30s_bio_12	original source	[37]
19	OlsenP kgha1 World	[home-folder]/XHEET_ASSETS/OlsenP_kgha1_World	original source	[38], [39]
20	Regridded Monthly Terrestrial Water Balance (University of Delaware)	[home-folder]/XHEET_ASSETS/Eo150_clim_xyz_updated	original source	[40]
21	Koppen-Geiger Global 1-km climate classification maps	[home-folder]/XHEET_ASSETS/Beck_KG_V1_present_0p0083	original source	[41]
22	HydroRIVERS v10	[home-folder]/HEET_ASSETS/HydroRIVERS_v10	original source	[42]
23	CCI Land Cover Maps (Years 1992, 2000, 2010, 2020)	[home-folder]/XHEET_ASSETS/ESACCI-LC-L4-LCCS-Map-300m-P1Y	original source, viewer	[43]

[1] `home-folder` is a placeholder for the private projects folder `projects/ee-future-dams/assets/` in Google Earth Engine.

[2] Source of hydrologically conditioned DEMs - 3 Arc-Seconds and 15 Arc-Seconds resolutions.

Supplementary Table 5: Input variables used by RE-Emission for estimating GHG emissions from reservoirs

Variable Name	Unit	Data source[1]
Inputs for catchment-level process calculations		
Biome	-	7
Climate	-	21
Soil Type	-	8
Treatment Factor[3]	-	–
Landuse Intensity[3]	-	–
Monthly Temperatures	^{o}C	17
Annual runoff	mm/year	16
Catchment area	km^2	2, 3, 4
Length of inundated river	km	22
Population	capita	14
Area fractions[2]	–	23
Mean catchment slope[5]	%	1 (5)
Mean annual precipitation[5]	mm/year	18
Mean annual evapotranspiration[5]	mm/year	20
Soil wetness	mm over profile[5]	13
Soil Olsen P content[5]	kgP ha^{-1}	19
Inputs for reservoir-level process calculations		
Reservoir volume[4]	m^3	
Reservoir area	km^2	1 (5)
Maximum reservoir depth[4]	m	
Mean reservoir depth[4]	m	
Inundated area fractions[1]	-	23
Soil carbon in inundated area	kgC m^{-2}	8
Mean monthly horizontal radiance	kWh m^{-2} d^{-1}	15
Mean monthly horizontal radiance: May - Sept	kWh m^{-2} d^{-1}	15
Mean monthly horizontal radiance: Nov - Mar	kWh m^{-2} d^{-1}	15
Mean monthly wind speed	m s^{-1}	12
Water intake depth below surface[3]	m	–

[1]Numbers refer to dataset indices in Table 4. In case alternative assets are available, they are given in parentheses.

[2]Fractions of land cover in the delineated area.

[3]Assumed or obtained from external sources.

[4]Reservoir volumes and mean and maximum depths need to be taken from external sources or derived from DEM (new reservoirs only).

[5]Inputs for an alternative phosphorus export estimation method [39], implemented in addition to the original phosphorus export calculations in the G-res model [44]

Supplementary Table 6: **Goodness-of-fit metrics for emissions estimated using Tier 1 emission factors compared to G-res model outputs [44].** The table reports the coefficient of determination (R^2), mean absolute error (MAE), and root mean squared error (RMSE) for four emission pathways: CO_2 diffusion, CH_4 diffusion, CH_4 ebullition, and CH_4 degassing. Results are shown for two sets of emission factors: global Tier 1 [19] (Original) and Myanmar-specific emission factors (Fitted) obtained via linear regression against emissions calculated with G-res.

Pathway	R^2		MAE		RMSE	
	EF (Original)	EF (Fitted)	EF (Original)	EF (Fitted)	EF (Original)	EF (Fitted)
CO_2 diffusion	-2.572	0.541	325.6	98.8	361.5	129.7
CH_4 diffusion	-2.160	0.304	208.2	90.6	239.6	112.5
CH_4 ebullition	-0.423	0.263	237.8	162.4	290.6	209.2
CH_4 degassing	-0.037	0.054	583.0	579.5	1108.5	1058.6

.

Supplementary Table 7: Net$_{CO_2}$ and net$_{CH_4}$ coefficients and the measures of fit between Tier 1 net emission estimates and G-res outputs. The net$_{CO_2}$ and net$_{CH_4}$ coefficients represent the fractions of anthropogenic (net) CO_2 and CH_4 emissions within gross (total) emissions, as expressed in Equation 1. The table reports the original coefficients published by Almeida et al. [20] (Original) alongside calibrated values (Fitted) obtained via linear regression, minimising the total squared error between Tier 1-based net emissions and the corresponding G-res estimates. Two sets of Tier 1 gross emission factors were used: global defaults [19] and Myanmar-specific factors derived by fitting to G-res gross emission outputs. Model performance is evaluated using three metrics: coefficient of determination (R^2), mean absolute error (MAE), and root mean squared error (RMSE). Margins of error in parentheses denote half-widths of 95% confidence intervals estimated via bootstrapping. All results assume a constant downstream emissions fraction of $R_{\text{downstream}}$ = 0.17 [20].

	Original				Fitted to net emission predictions from G-res							
					Global Tier 1 gross emission factors				Myanmar's Tier 1 gross emission factors			
	Value	R^2	MAE	RMSE	Value	R^2	MAE	RMSE	Value	R^2	MAE	RMSE
net$_{CO_2}$	0.250	0.543	3.495	13.6	0.378(0.081)	0.930	2.51	8.08	0.596(0.103)	0.955	2.164	6.558
net$_{CH_4}$	0.900	0.522	20.7	75.2	0.390(0.233)	0.401	12.2	36.5	0.404(0.237)	0.430	12.1	35.9

References

[1] Razavi, S. *et al.* The Future of Sensitivity Analysis: An essential discipline for systems modeling and policy support. *Environmental Modelling & Software* **137**, 104954 (2021). URL https://www.sciencedirect.com/science/article/pii/S1364815220310112.

[2] Lundberg, S. M. & Lee, S.-I. in *A Unified Approach to Interpreting Model Predictions* (eds Guyon, I. *et al.*) *Advances in Neural Information Processing Systems 30* 4765–4774 (Curran Associates, Inc., 2017). URL http://papers.nips.cc/paper/7062-a-unified-approach-to-interpreting-model-predictions.pdf.

[3] Stein, B. V. *et al.* A Comparison of Global Sensitivity Analysis Methods for Explainable AI With an Application in Genomic Prediction. *IEEE Access* **10**, 103364–103381 (2022).

[4] Razavi, S. & Gupta, H. V. What do we mean by sensitivity analysis? The need for comprehensive characterization of "global" sensitivity in Earth and Environmental systems models. *Water Resources Research* **51**, 3070–3092 (2015). URL https://agupubs.onlinelibrary.wiley.com/doi/abs/10.1002/2014WR016527.

[5] Molnar, C. *Interpretable Machine Learning* 2 edn (2022). URL https://christophm.github.io/interpretable-ml-book.

[6] Ali, S. *et al.* Explainable Artificial Intelligence (XAI): What we know and what is left to attain Trustworthy Artificial Intelligence. *Information Fusion* **99**, 101805 (2023). URL https://www.sciencedirect.com/science/article/pii/S1566253523001148.

[7] Gosiewska, A. & Biecek, P. Do Not Trust Additive Explanations (2020). 1903.11420.

[8] European Commission. Better regulation toolbox. WWW document) (2015). URL https://ec.europa.eu/info/law/law-making-process/planning-and-proposing-law/better-regulation-why-and-how/better-regulation-guidelines-and-toolbox/better-regulation-toolbox_en.

[9] Saltelli, A. *et al.* Five ways to ensure that models serve society: a manifesto. *Nature* **582**, 482–484 (2020).

[10] Razavi, S., Tolson, B. A. & Burn, D. H. Review of surrogate modeling in water resources. *Water Resources Research* **48** (2012). URL https://agupubs.onlinelibrary.wiley.com/doi/abs/10.1029/2011WR011527.

[11] Cheng, K., Lu, Z., Ling, C. & Zhou, S. Surrogate-assisted global sensitivity analysis: an overview. *Structural and Multidisciplinary Optimization* **61**, 1187–1213 (2020). URL https://doi.org/10.1007/s00158-019-02413-5.

[12] Quick, J. *et al.* Surrogate-based modeling and sensitivity analysis of future European electricity spot market prices. *Electric Power Systems Research* **234**, 110675 (2024). URL https://www.sciencedirect.com/science/article/pii/S0378779624005613.

[13] Ribeiro, M. T., Singh, S. & Guestrin, C. "Why Should I Trust You?": Explaining the Predictions of Any Classifier (2016). 1602.04938.

[14] Lundberg, S. M., Erion, G. G. & Lee, S.-I. Consistent Individualized Feature Attribution for Tree Ensembles (2019). URL https://arxiv.org/abs/1802.03888. 1802.03888.

[15] Flora, M., Potvin, C., McGovern, A. & Handler, S. Comparing Explanation Methods for Traditional Machine Learning Models Part 2: Quantifying Model Explainability Faithfulness and Improvements with Dimensionality Reduction (2022). URL https://arxiv.org/abs/2211.10378. 2211.10378.

[16] Monteiro, W. R. & Reynoso-Meza, G. A multi-objective optimization design to generate surrogate machine learning models in explainable artificial intelligence applications. *EURO Journal on Decision Processes* **11**, 100040 (2023). URL https://www.sciencedirect.com/science/article/pii/S2193943823000134.

[17] Masson-Delmotte, V. *et al.* Global warming of 1.5°C. An IPCC Special Report on the impacts of global warming of 1.5°C above pre-industrial levels and related global greenhouse gas emission pathways, in the context of strengthening the global response to the threat of climate change, sustainable development, and efforts to eradicate poverty. Tech. Rep., IPCC (2018).

[18] Li, M. & He, N. Carbon intensity of global existing and future hydropower reservoirs. *Renewable and Sustainable Energy Reviews* **162**, 112433 (2022). URL https://www.sciencedirect.com/science/article/pii/S1364032122003392.

[19] Soued, C., Harrison, J. A., Mercier-Blais, S. & Prairie, Y. T. Reservoir CO2 and CH4 emissions and their climate impact over the period 1900–2060. *Nature Geoscience* **15**, 700–705 (2022). URL https://doi.org/10.1038/s41561-022-01004-2.

[20] Almeida, R. M. *et al.* Reducing greenhouse gas emissions of Amazon hydropower with strategic dam planning. *Nature Communications* **10**, 4281 (2019). URL https://doi.org/10.1038/s41467-019-12179-5.

[21] Carlino, A., Schmitt, R., Clark, A. & Castelletti, A. Rethinking energy planning to mitigate the impacts of African hydropower. *Nature Sustainability* **7**, 879–890 (2024). URL https://doi.org/10.1038/s41893-024-01367-x.

[22] Tangi, M. *et al.* Robust Hydropower Planning Balances Energy Generation, Carbon Emissions and Sediment Connectivity in the Mekong River Basin. *Earth's Future* **12**, e2023EF003647 (2024). URL https://agupubs.onlinelibrary.wiley.com/doi/abs/10.1029/2023EF003647. E2023EF003647 2023EF003647.

[23] Kopec-Harding, K., Janus, T., Barry, C. & Kuriakose, J. GeoCARET: Geospatial Catchment and Reservoir analysis Tool. GitHub (2022). URL https://github.com/Reservoir-Research/geocaret.

[24] Janus, T., Barry, C. & Kuriakose, J. RE-Emission: A Python tool for calculating greenhouse gas emissions from reservoirs. GitHub (2022). URL https://github.com/tomjanus/reemission.

[25] Biecek, P. DALEX: Explainers for Complex Predictive Models in R. *Journal of Machine Learning Research* **19**, 1–5 (2018). URL http://jmlr.org/papers/v19/18-416.html.

[26] Baniecki, H., Kretowicz, W., Piatyszek, P., Wisniewski, J. & Biecek, P. dalex: Responsible Machine Learning with Interactive Explainability and Fairness in Python. *Journal of Machine Learning Research* **22**, 1–7 (2021). URL http://jmlr.org/papers/v22/20-1473.html.

[27] International Finances Corporation (IFC). SEA of the Hydropower Sector in Myanmar - Resources Page. Electronic (2018). URL https://www.ifc.org/en/insights-reports/2018/sea-of-the-hydropower-sector-in-myanmar-resources-page.

[28] Lehner, B., Verdin, K. & Jarvis, A. New Global Hydrography Derived From Spaceborne Elevation Data. *Eos, Transactions American Geophysical Union* **89**, 93–94 (2008). URL https://agupubs.onlinelibrary.wiley.com/doi/abs/10.1029/2008EO100001.

[29] Farr, T. G. *et al.* The Shuttle Radar Topography Mission. *Reviews of Geophysics* **45** (2007). URL https://agupubs.onlinelibrary.wiley.com/doi/abs/10.1029/2005RG000183.

[30] Dinerstein, E. *et al.* An Ecoregion-Based Approach to Protecting Half the Terrestrial Realm. *BioScience* **67**, 534–545 (2017). URL https://doi.org/10.1093/biosci/bix014.

[31] Poggio, L. *et al.* SoilGrids 2.0: producing soil information for the globe with quantified spatial uncertainty. *SOIL* **7**, 217–240 (2021). URL https://soil.copernicus.org/articles/7/217/2021/.

[32] Abatzoglou, J. T., Dobrowski, S. Z., Parks, S. A. & Hegewisch, K. C. TerraClimate, a high-resolution global dataset of monthly climate and climatic water balance from 1958–2015. *Scientific Data* **5**, 170191 (2018). URL https://doi.org/10.1038/sdata.2017.191.

[33] Chan, S. K. *et al.* Assessment of the SMAP Passive Soil Moisture Product. *IEEE Transactions on Geoscience and Remote Sensing* **54**, 4994–5007 (2016).

[34] Center for International Earth Science Information Network - CIESIN - Columbia University. Gridded Population of the World, Version 4 (GPWv4): Population Count, Revision 11. Palisades, New York: NASA Socioeconomic Data and Applications Center (SEDAC) (2019). Accessed 01 APR 2020.

[35] NASA Langley Atmospheric Sciences Data Center. Solar: Average Monthly and Annual Direct Normal Irradiance Data, One-Degree Resolution of the World from NASA/SSE, 1983-2005 (2008). [Shapefile]. Retrieved from https://earthworks.stanford.edu/catalog/stanford-fd535zg0917.

[36] Fekete, B. M., Vörösmarty, C. J. & Grabs, W. High-resolution fields of global runoff combining observed river discharge and simulated water balances. *Global Biogeochemical Cycles* **16**, 15–1–15–10 (2002). URL https://agupubs.onlinelibrary.wiley.com/doi/abs/10.1029/1999GB001254.

[37] Fick, S. E. & Hijmans, R. J. WorldClim 2: new 1-km spatial resolution climate surfaces for global land areas. *International Journal of Climatology* **37**, 4302–4315 (2017). URL https://rmets.onlinelibrary.wiley.com/doi/abs/10.1002/joc.5086.

[38] McDowell, R. Global Available Soil Phosphorus Database (2023). URL https://doi.org/10.6084/m9.figshare.14241854.v3. Dataset.

[39] McDowell, R. W., Noble, A., Pletnyakov, P. & Haygarth, P. M. A Global Database of Soil Plant Available Phosphorus. *Scientific Data* **10**, 125 (2023). URL https://doi.org/10.1038/s41597-023-02022-4.

[40] Willmott, C. J., Rowe, C. M. & Mintz, Y. Climatology of the terrestrial seasonal water cycle. *Journal of Climatology* **5**, 589–606 (1985). URL https://rmets.onlinelibrary.wiley.com/doi/abs/10.1002/joc.3370050602.

[41] Beck, H. E. *et al.* Present and future Köppen-Geiger climate classification maps at 1-km resolution. *Scientific Data* **5**, 180214 (2018). URL https://doi.org/10.1038/sdata.2018.214.

[42] Lehner, B. & Grill, G. Global river hydrography and network routing: baseline data and new approaches to study the world's large river systems. *Hydrological Processes* **27**, 2171–2186 (2013). URL https://onlinelibrary.wiley.com/doi/abs/10.1002/hyp.9740.

[43] Copernicus Climate Change Service. Land cover classification gridded maps from 1992 to present derived from satellite observation. Climate Data Store, Copernicus Climate Change Service (C3S) Climate Data Store (CDS) (2019). URL https://cds.climate.copernicus.eu/cdsapp#!/dataset/satellite-land-cover?tab=form.

[44] Prairie, Y. T. *et al.* A new modelling framework to assess biogenic GHG emissions from reservoirs: The G-res tool. *Environmental Modelling & Software* **143**, 105117 (2021). URL https://www.sciencedirect.com/science/article/pii/S1364815221001602.
